# Evaluation of ventilatory parameters reporting in large animal models of cardiac arrest: a scoping review^☆^

**DOI:** 10.1016/j.resplu.2025.101185

**Published:** 2025-12-08

**Authors:** Jean-Claude Li, Nicolas Segond, Arnaud Lesimple, Alice Hutin, Rebecca Goutchtat, Iann Drennan, Giuseppe Ristagno, Guillaume Debaty, Alain Cariou, Renaud Tissier, Jean-Christophe Richard

**Affiliations:** a Ecole nationale vétérinaire d’Alfort, IMRB, AfterROSC Network, 94700 Maisons-Alfort, France; b University Paris Est Créteil, INSERM, IMRB, F-94010 Créteil, France; c Medical ICU, Cochin Hospital, AP-HP Centre Université Paris Cité, AfterROSC Network, Paris, France; d Emergency Department and Mobile Intensive Care Unit, University Hospital of Grenoble Alpes, Grenoble, France; e Univ. Grenoble Alpes, CNRS, UMR 5525, VetAgro Sup, Grenoble INP, TIMC, 38000 Grenoble, France; f Vent’Lab, Medical Intensive Care Unit, Angers University Hospital, Angers, France; g Med_2_Lab Laboratory, Air Liquide Medical Systems, Antony, France; h SAMU of Paris and Intensive Care Unit, Necker University Hospital, Assistance Publique-Hôpitaux de Paris (APHP), Paris 75015, France; i Department of Family and Community Medicine, Division of Emergency Medicine, University of Toronto, Toronto, Ontario, Canada; j Department of Pathophysiology and Transplantation, University of Milan, Italy; k Department of Anesthesiology, Intensive Care and Emergency, Fondazione IRCCS Ca’ Granda Ospedale Maggiore Policlinico, Milan, Italy

**Keywords:** Cardiac arrest, Cardiopulmonary resuscitation, Ventilation, Ventilator, Tidal volume, Animal models, Manual ventilation

## Abstract

**Background:**

Ventilation is a critical determinant of cardiopulmonary resuscitation efficiency. Our goal was to evaluate how ventilatory parameters are reported during cardiopulmonary resuscitation in large animal models of cardiac arrest.

**Methods:**

A scoping review was conducted following the PRISMA-ScR guidelines, including studies referenced in Pubmed over the last decade (January 1st, 2015 to July 30th, 2025). The review followed the PCC approach: (P) population: large animal models of cardiac arrest; (C) concept: ventilatory settings and parameters during CPR; (C) context: studies aiming at describing or evaluating mechanical or manual ventilation during CPR in experimental conditions. The reporting of the animal characteristics, ventilatory settings and monitored parameters were extracted and analyzed descriptively.

**Results:**

We identified 111 relevant publications. Most of them used porcine models (79 %), with ventricular fibrillation being the most common method of cardiac arrest induction (59 %). Mechanical ventilation was predominant (75 %), with volume and pressure-controlled modes nearly equally represented. The reporting of critical ventilatory settings was inconsistent, with a percentage of appropriate reporting as follows: respiratory rate (88 %), fraction of inspired oxygen (83 %), positive end-expiratory pressure (49 %), tidal volume (83 %, among studies with volume-controlled ventilation), peak inspiratory pressure (92 %, among studies with pressure-controlled ventilation) and inspiratory to expiratory ratio (17 %, among all studies with mechanical ventilation). Reporting of measured ventilatory parameters during CPR was also limited with, e.g., EtCO_2_ reported in 41 % of the studies and arterial blood gases sampled and reported in 50 % of the studies.

**Conclusions:**

This scoping review evidenced substantial variability and frequent omissions in the reporting of ventilatory settings and monitoring in large animal CPR studies. Updated recommendations could be useful to provide specific guidelines of reporting in the field.

## Introduction

In the field of resuscitation research, large animal models are essential for the translation of experimental findings to clinical practice. In particular, pigs closely mimic human cardiovascular and pulmonary physiology,^1^, ^2^ allowing for realistic modeling of ventilatory and hemodynamic interactions during cardiopulmonary resuscitation (CPR). Beyond chest compression, which has been extensively investigated over the last decades, the impact of ventilation during CPR is critical, but still insufficiently studied. For instance, many ventilatory parameters such as tidal volume (Vt) or positive end-expiratory pressure (PEEP) are known to influence intrathoracic pressure, coronary perfusion pressure and the likelihood of return of spontaneous circulation (ROSC).^3^, ^4^, ^5^, ^6^ When insufficient, ventilation may impair gas exchange and limit oxygenation, while excessive ventilation could compromise circulation by increasing lung volume above functional residual capacity (FRC) and negatively impact venous return.^7^ In addition, the continuous monitoring of ventilatory parameters can provide surrogate markers of perfusion and ventilation quality during CPR with e.g., end-tidal CO_2_ (EtCO_2_) or full capnogram analysis.^8^, ^9^ Accordingly, rigorous reporting of experimental protocols and ventilatory parameters is essential in CPR animal studies to ensure reproducibility and translational relevance in preclinical research.

In line with these requirements, the ARRIVE 2.0 guidelines, updated in 2020, offered a structured checklist for the general report of animal studies.^10^ However, these guidelines only established general standards. More specifically, the Utstein-Style guidelines published in 1996^11^ proposed a first template to harmonize resuscitation data reporting, including ventilatory support, for laboratory CPR research. It remains unclear whether these recommendations have effectively improved consistency across experimental studies, particularly in light of the recent progress in ventilation and monitoring strategies during CPR.

Accordingly, this scoping review aimed at systematically evaluating how ventilatory parameters were reported in large-animal CPR studies over the last decade. Our literature search strategy focused on studies properly describing and evaluating ventilation during CPR. The goal is to pave the way for up-to-date future recommendations regarding ventilatory monitoring and reporting in large animals CPR studies.

## Methods

### Design

This scoping review was drafted following the requirements of the Preferred Reporting Items for Systematic reviews and Meta-Analyses extension for Scoping Review (PRISMA-ScR).

### Eligibility criteria

This scoping review included studies which matched the following PCC approach: (P) population: defined as large animal models of cardiac arrest; (C) concept: defined as ventilatory settings and monitored parameters during CPR; (C) context: experimental laboratory studies on large animals, describing mechanical or manual ventilation under controlled CPR conditions. The search was restricted to English publications. The following types of publication were excluded: case reports, reviews, letters, commentaries, editorials, comments. Animal models including the use of extracorporeal circulation were also excluded.

### Information source

Considering that ventilatory management during CPR is constantly evolving both in clinical and preclinical studies, the search was restricted to the past ten years to ensure that the included studies reflect current animal experimental practices. The bibliographic database PubMed was searched for articles published from January 1st, 2015 to July 30th, 2025.

### Search strategy and selection process

The search strategy was developed and refined through team discussions. It contained keywords relevant to ventilation during cardiopulmonary resuscitation in large animal models of cardiac arrest. The full literature search strategy is provided in Supplemental material.

Articles were initially screened based on their titles and abstracts. Following the initial screening, relevant full-text articles identified were reviewed. Any doubt regarding the inclusion or exclusion of an article was resolved by discussion with a second independent reviewer.

### Data charting and collection

A data-charting form was developed by team discussion to determine which variables to extract. Two reviewers discussed the results and updated the data-charting form in an iterative process. Data extracted from the selected papers were classified into 3 categories: (1) Characteristics of animal models of CPR during cardiac arrest, (2) Ventilatory settings during CPR, and (3) Ventilatory parameters monitored and reported during CPR.

Animal model characteristics included: the total number of animals, body weight, sex, method of cardiac arrest induction, and the modality and rate of chest compressions.

Ventilatory settings reported during CPR included: ventilation interface and modality (mechanical or manual), mode of ventilation when mechanical, ventilatory settings such as respiratory rate, fraction of inspired oxygen (FiO_2_) or oxygen flow (for mechanical and manual ventilation respectively), tidal volume (Vt) and PEEP. Settings specific to mechanical ventilation including inspiratory trigger, inspiratory to expiratory ratio (I:E), and peak pressure alarm, as well as settings specific to manual ventilation including pressure-release valve and bag volume were also investigated.

**Fig. 1:**
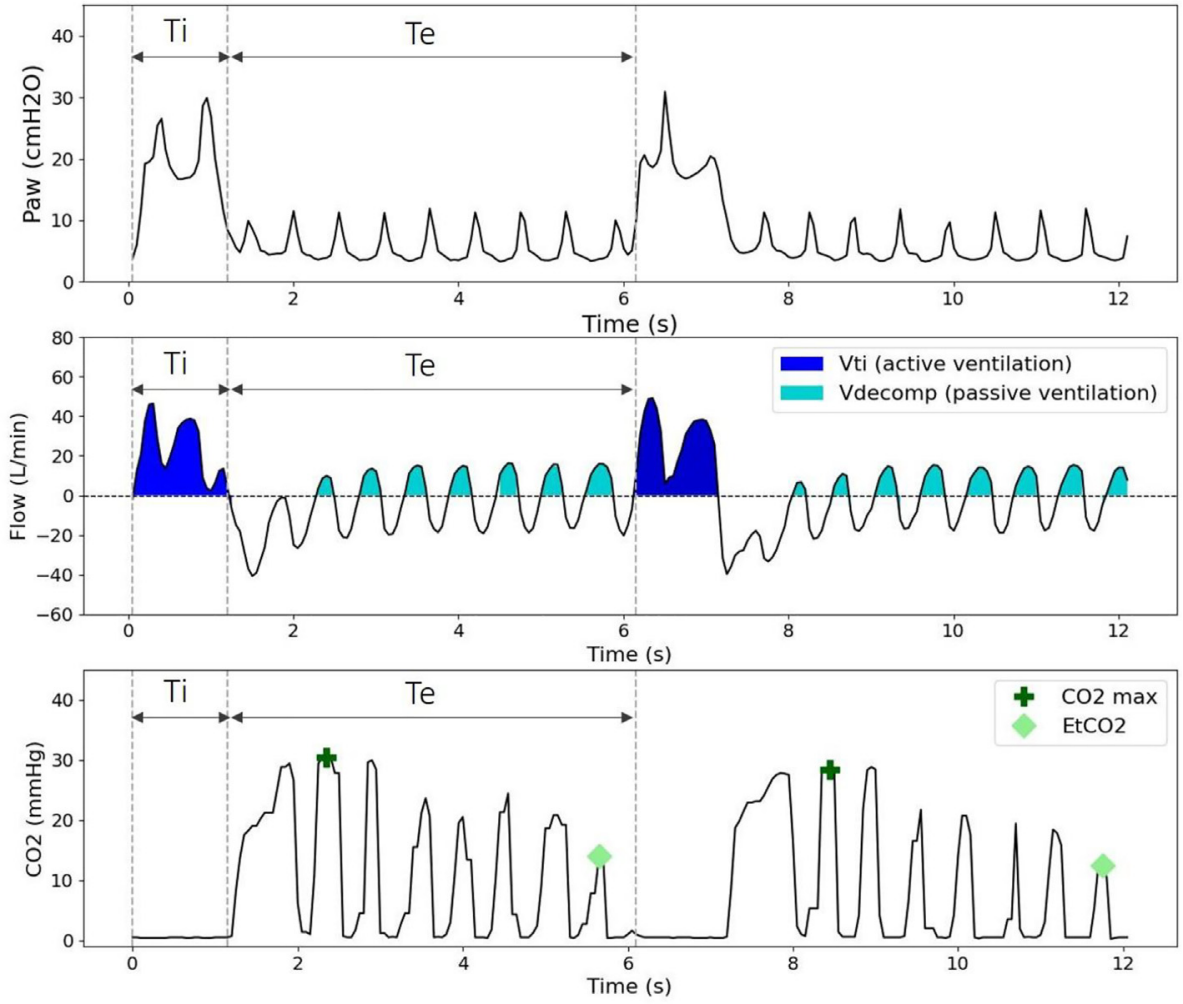
**Schematic representation of the waveforms for important ventilation parameters during cardiopulmonary resuscitation**. *Paw, Airway pressure; Ti, inspiration time; Te, expiration time; Flow, airway flow; Vti, inspiratory tidal volume, corresponding to the usual Vt value (active ventilation); Vdecomp, passive ventilation during chest decompression; CO_2_ max, maximal exhaled CO_2_ during expiration; EtCO_2_, end-tidal CO_2_*.

Ventilatory parameters monitored during CPR included: Vt, ventilation induced by chest compressions called passive ventilation (Fig. 1), minute volume, peak inspiratory pressure, intrathoracic pressure, and gas exchange variables such as peripheral oxygen saturation (SpO_2_), end-tidal carbon dioxide (EtCO_2_) with or without volumetric analysis, and arterial blood gases. Fig. 1 also schematically illustrates airway pressures and full capnogram during CPR.

### Data synthesis and analysis

Variables were presented as numbers and percentages of publications evaluating or reporting each condition or parameter. Numerical values of set parameters during CPR were expressed as median and interquartile range. Statistical analyses were performed with Microsoft Excel Software©.

## Results

### Study selection

**Fig. 2:**
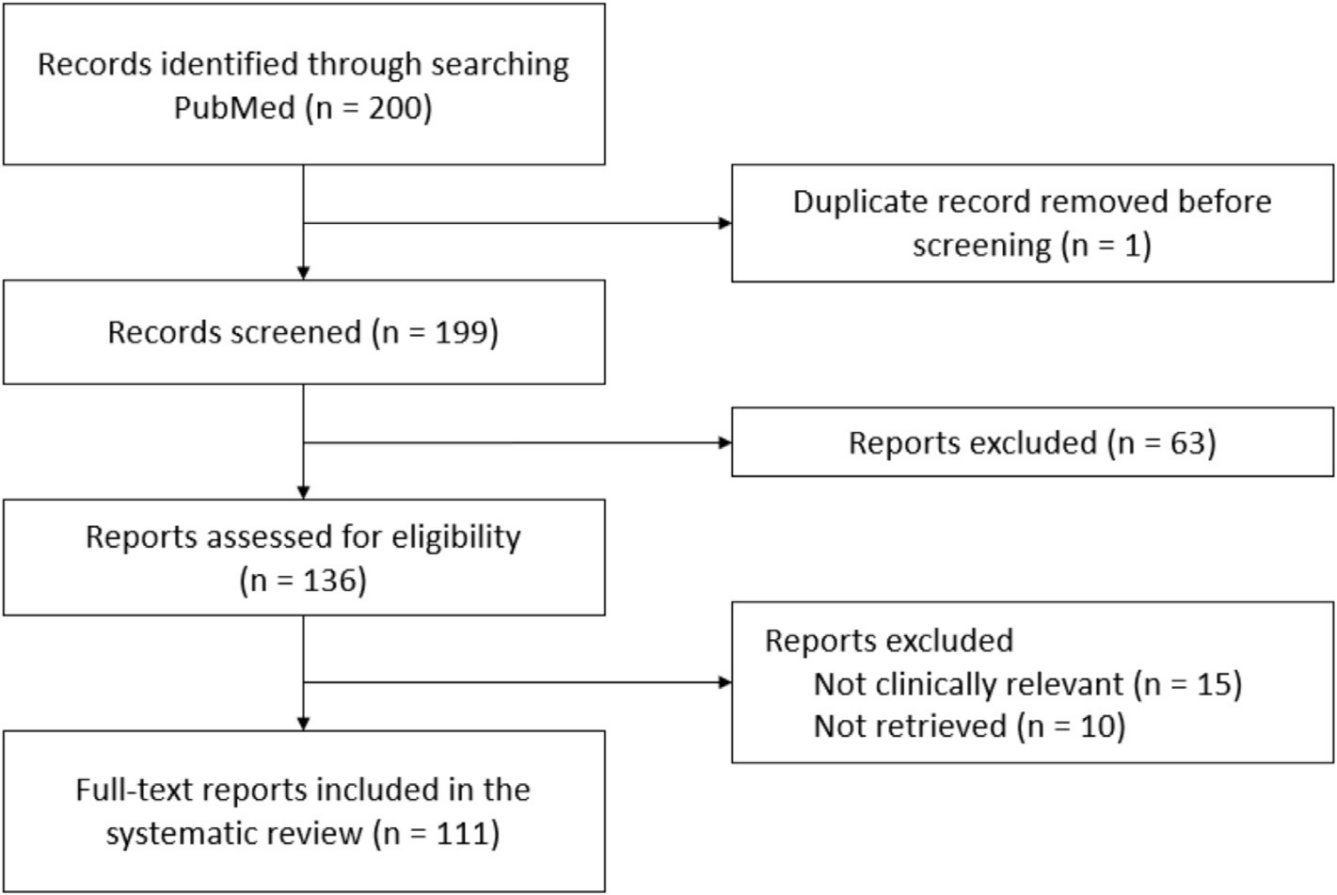
**Flow chart of study selection**.

A total of 200 studies were identified from PubMed. After excluding one corrigendum counted as a duplicate, 199 studies were screened by title and abstract. Out of these, 126 studies passed the first screening and had full-text articles retrieved. Following secondary screening, 15 were excluded because the experimental configuration was not considered clinically relevant to ventilation monitoring. The remaining 111 studies were included in the final analysis. The agreement between reviewers during the screening process was assessed to ensure consistency in study selection, resulting in a Cohen’s kappa coefficient of 0.8, indicating excellent agreement. Fig. 2 shows the PRISMA flow diagram for study inclusion and exclusion.

### Animal characteristics

As shown in Table 1, the vast majority of studies employed a porcine model (79 %), and the remainder used lamb (16 %), or canine (5 %) models. The median number of animals per study was 22 (IQR 13–32). Median body weight was 32 kg (IQR 12–39) in pigs, 4 kg (IQR 4–5) in lambs and 25 kg (IQR 13–27) in dogs. Lamb studies mostly focused on pediatric conditions (4–5 kg). They were included in the full analysis as complying in the initial search strategy. When reported, sex distribution was relatively balanced, with 21 % males, 19 % females, and 36 % including both sexes.

**Table 1:** Main characteristics of the animal models in the included studies. Data are expressed as mean and IQR or number of publications and percentage of total number for each species.

	**Total (*N* = 111)**	**Pig (*N* = 88)**	**Lamb (*N* = 18)**	**Dog (*N* = 5)**
Number of animals	22 (13–32)	20 (12–32)	24 (18–33)	20 (12–24)
Weight (kg)	27 (5–36)	32 (12–39)	4 (4–5)	25 (13–27)

**Sex**				
Male	23 (21 %)	23 (26 %)	0 (0 %)	4 (80 %)
Female	21 (19 %)	17 (19 %)	0 (0 %)	1 (20 %)
Both	40 (36 %)	21 (24 %)	18 (100 %)	0 (0 %)
Not reported	27 (24 %)	27 (31 %)	0 (0 %)	0 (0 %)

**Cardiac arrest**				
Ventricular fibrillation	66 (59 %)	62 (70 %)	0 (0 %)	4 (80 %)
Asphyxia	43 (39 %)	24 (27 %)	18 (100 %)	1 (20 %)
Other	2 (2 %)	2 (2 %)	0 (0 %)	0 (0 %)

Chest compressions modality*				
Mechanical	58 (52 %)	57 (65 %)	0 (0 %)	1 (20 %)
Manual	39 (35 %)	30 (34 %)	5 (28 %)	4 (80 %)
*Quality monitoring*	*8/39 (21 %)*	*8/30 (27 %)*	*0/5 (0 %)*	*0/4 (0 %)*
Not reported	17 (15 %)	4 (5 %)	13 (72 %)	0 (0 %)

Chest compressions*				
Continuous	75 (68 %)	72 (82 %)	3 (17 %)	0 (0 %)
Interrupted	39 (35 %)	22 (25 %)	14 (78 %)	3 (60 %)
Not reported	10 (9 %)	7 (8 %)	1 (6 %)	2 (40 %)

* For chest compressions (mechanical vs manual or continuous vs interrupted), percentage can be superior to 100 % since some studies compared different conditions*.*

In most studies, cardiac arrest was induced by ventricular fibrillation with electrical stimulation (59 %). Asphyxia, achieved by occluding the endotracheal tube and disconnecting the ventilator, constituted the second most frequent method (39 %).

The majority of studies used chest compressions with an automated mechanical device (52 %). When manual chest compressions were applied (35 %), compression quality was monitored in 21 % of the studies. Continuous chest compressions were more frequently reported (68 %) than interrupted compressions (35 %), considering that few studies compared both conditions and were included in both categories.

### Ventilatory settings during cardiopulmonary resuscitation

As shown in Table 2, almost all studies relied on invasive ventilation (95 %), most frequently with mechanical ventilation (75 %), while manual bag-valve ventilation was reported in 26 % of the studies. Continuous-flow oxygen was rarely used (6 %).

**Table 2:** Reporting of the ventilatory settings during cardiopulmonary resuscitation (CPR) in the included studies.

	**Total (*N* = 111)**	**Pig (*N* = 88)**	**Lamb (*N* = 18)**	**Dog (*N* = 5)**
**Ventilation**				
Invasive	106 (95 %)	85 (97 %)	18 (100 %)	3 (60 %)
Non-invasive	1 (1 %)	1 (1 %)	0 (0 %)	0 (0 %)
Both	4 (4 %)	2 (2 %)	0 (0 %)	2 (40 %)

Ventilation modality*				
Mechanical	83 (75 %)	61 (69 %)	18 (100 %)	4 (80 %)
Bag valve device	29 (26 %)	27 (31 %)	1 (6 %)	1 (20 %)
Continuous flow oxygen	7 (6 %)	7 (8 %)	0 (0 %)	0 (0 %)
Not reported	3 (3 %)	3 (3 %)	0 (0 %)	0 (0 %)

Ventilation mode (when mechanical)*				
Volume-controlled	40/83 (48 %)	39/61 (64 %)	0/18 (0 %)	1/4 (25 %)
*Report of Vt set point*	*33/40 (83 %)*	*33/39 (85 %)*	–	*0/1 (0 %)*
*Report of Vt relatively to body weight*	*30/40 (75 %)*	*30/39 (77 %)*	–	*0/1 (0 %)*
Pressure-controlled	38/83 (46 %)	20/61 (33 %)	18/18 (100 %)	0/4 (0 %)
*Report of inspiratory pressure set point*	*35/38 (92 %)*	*18/20 (90 %)*	*17/18 (94 %)*	–
Specific CPR mode (e.g., CPV, CCSV, ULTVV)	9/83 (11 %)	9/61 (15 %)	0/18 (0 %)	0/4 (0 %)
Not reported	11/83 (13 %)	8/61 (13 %)	0/18 (0 %)	3/4 (75 %)

**Report of other ventilation parameters**				
Respiratory rate	98 (88 %)	77 (88 %)	18 (100 %)	3 (60 %)
FiO_2_ or Oxygen flow	92 (83 %)	70 (80 %)	18 (100 %)	4 (80 %)
PEEP	54 (49 %)	37 (42 %)	17 (94 %)	0 (0 %)

***Among studies with mechanical ventilation***				
*Inspiratory trigger*	5/83 (6 %)	5/61 (8 %)	0/18 (0 %)	0/4 (0 %)
*I:E ratio*	14/83 (17 %)	12/61 (20 %)	2/18 (11 %)	0/4 (0 %)
*Pressure alarm*	5/83 (6 %)	5/61 (8 %)	0/18 (0 %)	0/4 (0 %)

***Among studies with bag valve device***				
*Pressure release valve*	3/29 (10 %)	3/27 (11 %)	0/1 (0 %)	0/1 (0 %)
*Bag volume*	1/29 (3 %)	1/27 (4 %)	0/1 (0 %)	0/1 (0 %)

Data are expressed as number of publications using or reporting the corresponding setting or parameter, as well as percentage of publication number.

Vt, tidal volume; Ppeak, peak inspiratory pressure; CPV, cardiopulmonary ventilation; CCSV, chest compression synchronized ventilation; ULTVV, ultra-low tidal volume ventilation; PEEP, positive end-expiratory pressure; FiO_2_, fraction of inspired oxygen.

* For ventilation modality and mode, percentage can be superior to 100% since some studies compared different conditions.

Among mechanical ventilation strategies, volume-controlled (48 %) and pressure-controlled (46 %) modes were nearly equally represented. Among the studies with volume or pressure-controlled ventilation, a small proportion of studies reported specific CPR-adapted modes (11 %), including ultra-low tidal volume ventilation (ULTVV, 5 %), chest compression synchronized ventilation (CCSV, 4 %), or cardiopulmonary ventilation (CPV, 1 %).

Among ventilatory settings, the most frequently documented parameters were respiratory rate (88 %), Vt (83 % among studies with volume-controlled ventilation), inspiratory pressure (92 %, among studies with pressure-controlled ventilation), FiO_2_ or oxygen flow (83 %), and PEEP (49 %). In contrast, only few studies reported the settings (specific to mechanical ventilation) for inspiratory to expiratory ratio (17 %), inspiratory trigger (6 %), or inspiratory pressure alarm (6 %). Numerical values of the corresponding set parameters are shown in Table 3.

**Table 3:** Numerical values of set parameters during cardiopulmonary resuscitation (CPR) in the included studies when corresponding values were available.

	**Total**	**Pig**	**Lamb**	**Dog**
Respiratory rate (/per minute)*	10 (10–30) *N* = 93	10 (10–20) *N* = 73	40 (30–40) *N* = 17	7 (7–9) *N* = 3
FiO_2_ (%)†	100 (100–100) *N* = 83	100 (100–100) *N* = 61	100 (21–100) *N* = 18	61 (21–100) *N* = 4
PEEP (cmH_2_O)	5 (1–5) *N* = 54	5 (0–5) *N* = 37	5 (5–5) *N* = 17	*N* = 0
I:E ratio	1:2 (1:1.4–1:2) *N* = 14	1:2 (1:1.3–1:2) *N* = 12	1:2 (1:2–1:2) *N* = 2	*N* = 0
Pressure alarm (cmH_2_O)	60 (60–60) *N* = 5	60 (60–60) *N* = 5	*N* = 0	*N* = 0
Set Vt (mL/kg) among studies with volume-controlled ventilation‡	10 (7–10) *N* = 33	10 (7–10) *N* = 33	*N* = 0	*N* = 0
Targeted Vt mL/kg, among studies with bag-valve device‡	9 (8–10) *N* = 19	9 (8–10) *N* = 18	*N* = 0	12 (12–12) *N* = 1
Inspiratory pressure (cmH_2_O) among studies with pressure-controlled ventilation	30 (25–34) *N* = 35	25 (20–30) *N* = 18	33 (30–35) *N* = 17	*N* = 0

When different values were reported in the same study in different groups, each experimental group was counted independently. Data are expressed as median (IQR).

FiO_2_, fraction of inspired oxygen; PEEP, positive end-expiratory pressure; Vt, tidal volume.

* We couldn’t calculate the respiratory rate for three studies as they only reported the compression to ventilation ratio without reporting the compression rate. We also excluded studies with continuous insufflation.

† We excluded studies which reported oxygen flow and not fraction of inspired oxygen.

‡ When tidal volume was not indexed to body weight, we reported it to mean weight of animals in the corresponding study.

### Reporting of ventilatory parameters during cardiopulmonary resuscitation (CPR)

Table 4 shows the reporting of the different ventilatory parameters monitored during CPR and reported in the included studies. Among all studies, only 23 % reported Vt, 22 % documented peak inspiratory pressure, and 11 % minute volume. Additionally, passive ventilation (3 %) and intrathoracic pressure (3 %) were rarely measured.

**Table 4:** Reporting of monitored ventilatory parameters during cardiopulmonary resuscitation (CPR) in the included studies.

	**Total (*N* = 111)**	**Pig (*N* = 88)**	**Lamb (*N* = 18)**	**Dog (*N* = 5)**
**Evaluation and report of monitored ventilation parameters**				
Vt	25 (23 %)	21 (24 %)	4 (22 %)	0 (0 %)
*Inspiratory Vt*	*8/25 (32 %)*	*8/21 (38 %)*	*0/4 (0 %)*	–
*Expiratory Vt*	*7/25 (28 %)*	*7/21 (33 %)*	*0/4 (0 %)*	–
Minute volume	12 (11 %)	12 (14 %)	0 (0 %)	0 (0 %)
Passive ventilation	3 (3 %)	3 (3 %)	0 (0 %)	0 (0 %)
Ppeak	24 (22 %)	24 (27 %)	0 (0 %)	0 (0 %)
Intrathoracic pressure	3 (3 %)	3 (3 %)	0 (0 %)	0 (0 %)

**Evaluation and report of gas exchange parameters (during CPR)**				
EtCO_2_	45 (41 %)	44 (50 %)	0 (0 %)	1 (20 %)
*Volumetric capnography*	*7/45 (16 %)*	*7/44 (16 %)*	–	*0/1 (0 %)*
*Mainstream sensor*	*10/45 (22 %)*	*10/44 (23 %)*	–	*0/1 (0 %)*
*Side stream sensor*	*2/45 (4 %)*	*2/44 (5 %)*	–	*0/1 (0 %)*
*Not reported*	*29/45 (64 %)*	*28/44 (64 %)*	–	*1/1 (100 %)*
Blood gases (per CPR)	56 (50 %)	42 (48 %)	11 (61 %)	3 (60 %)

**Evaluation of arterial and tissue oxygenation (during CPR)**				
SpO_2_	4 (4 %)	4 (5 %)	0 (0 %)	0 (0 %)
SaO_2_	15 (14 %)	10 (11 %)	4 (22 %)	1 (20 %)
SvO_2_ or PvO_2_	14 (13 %)	12 (14 %)	1 (6 %)	1 (20 %)
Regional cerebral oxygenation	26 (23 %)	22 (25 %)	4 (22 %)	0 (0 %)

Importantly, measured parameters can differ from set parameters due to metrological and physiological reasons, emphasizing the importance of subsequent reporting (e.g., Vt during bag ventilation, etc).

Data are expressed as numbers of publication using or reporting the corresponding setting or parameter, as well as percentage of publication number.

Vt, tidal volume; Ppeak, peak inspiratory pressure; EtCO_2_, end-tidal CO_2_; SpO_2_, peripheral capillary oxygen saturation; SaO_2_, arterial oxygen saturation; SvO_2_, mixed venous oxygen saturation; PvO_2_, mixed venous oxygen pressure.

EtCO_2_ monitoring during CPR was described in 41 % of the studies. Only 26 % specified the sensor type (mainstream or sidestream), and 16 % reported the use of volumetric capnography. Arterial blood gases were sampled during CPR in 50 % of the studies. Of note, 12 studies mentioned EtCO_2_ monitoring during CPR without reporting the corresponding data, 9 studies monitored peak inspiratory pressure but did not report it, and 4 studies monitored Vt and minute ventilation without providing the associated values.

Evaluation of arterial and/or tissular oxygenation was rare as only 14 % of the studies reported arterial oxygen saturation (SaO_2_), 13 % reported mixed venous oxygen saturation (SvO_2_) or mixed venous oxygen pressure (PvO_2_) as indicators of tissue oxygen consumption, and 23 % reported regional cerebral oxygenation with the use of near infrared spectroscopy or direct intraparenchymal probe.

## Discussion

In this scoping review, we provided a comprehensive overview of ventilatory parameters reporting in large animal models of CPR over the past decade, thirty years after the establishment of the Utstein-Style Guidelines.^11^ To our knowledge, this is the first review to systematically describe ventilatory settings and monitoring practices during experimental CPR.

Our analysis yielded several key observations. First, our findings are consistent with previous reports highlighting variability in ventilatory strategies during both clinical and experimental CPR.^12^ Indeed, ventilatory strategies and settings were highly heterogenous across studies. Most of the basic data recommended by the Utstein-Style Guidelines were correctly reported: ventilatory interface (100 %), ventilation mode when mechanical (88 %), inspired O_2_ concentration or oxygen flow (83 %), Vt set (83 %), respiratory rate (88 %), synchronization with chest compressions (91 %). The description of the PEEP level (49 %) and the inspiration to expiration ratio when mechanical (17 %) were frequently neglected. Finally, specific ventilator settings of inspiratory trigger and peak inspiratory pressure alarm were almost never documented. In addition, monitoring of many variables were often absent, such as Vt during CPR (23 %), minute volume (11 %) or peak inspiratory pressure (22 %). In fact, each of these parameters has the potential to significantly influence delivered ventilation that may impact intrathoracic pressure, hemodynamics, and ultimately the general outcome of most studies. Second, despite clinical findings and Utstein-Style guidelines emphasizing the importance of monitoring EtCO_2_ and blood gases as surrogates for perfusion and ventilation quality,^11^, ^13^ these variables were underreported in our review. EtCO_2_ during CPR was described in less than half of the studies, and when available, waveform analysis or volumetric capnography was rarely included despite their potential additional value.^14^, ^15^ Similarly, few studies documented intrathoracic pressure, even though it is highly relevant for assessing interactions between ventilation and chest compressions during CPR.^16^

This lack of standardization has significant implications. Incomplete or heterogeneous reporting limits comparison across studies, reduces reproducibility, and hinders the interpretation of experimental findings. Furthermore, given the increasing reliance on animal models to guide clinical practice, harmonized reporting of ventilatory settings and monitoring during CPR is essential, along with the consistent use of a common terminology as previously suggested.^17^

For instance, even if the settings for Vt were among the most widely reported in our analysis, the monitoring of the actual measured inspiratory and expiratory Vt was very rare. Moreover, no methodological recommendations currently exist regarding its measurement. The optimal Vt to be delivered during CPR, i.e. active ventilation, remains debated. In the clinical field, a recent post hoc analysis of a large randomized controlled trial even suggested that ventilation, measured by thoracic impedance, is most of the time inadequate before advanced airway placement, and likely associated with a worse prognosis.^18^ Therefore, measuring and reporting of Vt is essential. Interestingly, after intubation, inspiratory Vt (Vt delivered during inspiration time) should be preferred, as expiratory Vt is affected by chest compressions and their impact on absolute lung volume (Fig. 1). Of note, monitoring Vt (whether inspiratory or expiratory) during ventilation without a secured airway remains highly challenging due to inspiratory and expiratory leaks at the mask interface.^19^, ^20^ Ventilation that results from repeated chest compressions and decompressions during the expiration time, usually called passive ventilation, may also contribute to overall gas exchange. In the landmark study from Peter Safar, passive ventilation evaluated in humans was highly variable with gas movements induced by chest compressions ranging from 0 ml to more than 50 ml.^21^ In addition, the recently described airway closure phenomenon has been incriminated as limiting passive ventilation.^22^ Interestingly, internal functioning of different medical devices may significantly increase (e.g. active decompression) or stop passive ventilation (e.g. impedance threshold device or PEEP valve), thus impacting gas exchange and absolute lung volumes. For this purpose, the measurement of flow entering into the thorax during decompression (i.e., expiratory reverse flow, also known as ventilation volume during decompression) may be recommended as a surrogate of passive ventilation (Fig. 1).

Among the monitored parameters acting as possible surrogates of ventilation and CPR efficiency, it was also surprising that EtCO_2_ was not widely reported. Although EtCO_2_ monitoring is easily available in laboratory settings, its proper interpretation could remain challenging. EtCO_2_ variation could indeed reflect blood flow induced by chest compressions but also changes of global ventilation. A previous work showed that rather than the EtCO_2_ value, the maximum value of exhaled CO_2_ (CO_2_ max) might be a closer surrogate of alveolar CO_2_, and should therefore be monitored (Fig. 1).^23^ Thus, caution is warranted when interpreting CO_2_ signal. More recently, specific CO_2_ patterns have been described allowing to recognize potentially harmful situations such as airway closure or thoracic distension; in these cases, adapting ventilation settings may permit to optimize respiratory support.^15^ The analysis of the capnogram could be considered as an insightful parameter to report in complement of EtCO_2_ measurements (including sensor type).

Accordingly, as a minimal requirement, future studies should ensure consistent reporting of the parameters recommended by the current Utstein-Style guidelines, including animal characteristics (weight, sex, method of cardiac arrest induction), ventilatory interface and mode (when mechanical ventilation), FiO_2_ or oxygen flow, Vt, PEEP, respiratory rate, and I:E ratio. During CPR, peak inspiratory pressure, inspiratory Vt, real minute volume, EtCO_2_ and blood gas sampling with arterial and mixed venous oxygen saturation should also be documented. Additionally, we believe that it is important to report indexed inspiratory Vt to body weight and inspiratory trigger setting for mechanical ventilation. As stated above, measurement of expiratory Vt (when airways are secured) should not be recommended as it is highly dependent upon ventilators or monitoring devices and largely heterogenous due to the impact of chest compressions. We also encourage the documentation of intrathoracic pressure, which could provide relevant information to interpret the potential hemodynamic impact of ventilation. To support this purpose, we provided a structured template of key variables that appear important for standardized reporting in future experimental CPR protocols (Table 5). Of note, the direct assessment of the hemodynamic effects of ventilation during CPR (which is not within the scope of this work) also provide valuable insights (e.g., using coronary perfusion pressure and tissular oxygen delivery).

**Table 5:** Proposition of ventilation settings and parameters that should be reported and measured for a comprehensive description of ventilation impact in large animal models of cardiopulmonary resuscitation (CPR).

**Ventilatory settings during CPR**	
Interface	Invasive or non-invasive
Modality	Mechanical, bag valve device, continuous flow oxygen or other
Mode	Volume-controlled, pressure-controlled, or specific CPR mode
Ventilation parameters	Respiratory rate, FiO_2_, Vt (reported to body weight), PEEP, inspiratory trigger, and I:E ratio

**Ventilatory parameters monitored during CPR**	
Ventilation monitoring	Inspiratory Vt (active ventilation), Ppeak, minute volume (including passive ventilation), Source of measurements (ventilator or laboratory recordings) *N.B.: Ideally, intrathoracic pressure is relevant during mechanical ventilation, but its measurement during CPR is technically difficult because the dynamic and unstable conditions complicate accurate and reproducible assessment*
Gas exchange parameters	Blood gases EtCO_2,_ sensor type and ideally volumetric or waveform analysis (e.g., CO_2_ max)
Arterial and tissular oxygenation	SaO_2_, SvO_2_ and/or PvO_2_ Ideally, cerebral oximetry with near infrared spectroscopy or intraparenchymal probe

FiO_2_, fraction of inspired oxygen; Vt, tidal volume; PEEP, positive end-expiratory pressure; Ppeak, peak inspiratory pressure; EtCO_2_, end-tidal CO_2_; SaO_2_, arterial oxygen saturation; SvO_2_, mixed venous oxygen saturation; PvO_2_, mixed venous oxygen pressure.

This scoping review has several limitations. First, our review was limited to English-language publications over the past 10 years. Second, the heterogeneity of protocols prevented quantitative synthesis and the reliability of our analysis depended on the extent of reporting provided in the included studies. Finally, our review was restricted to large animal models, which may limit generalizability to small species or clinical settings. Although large animal models provide valuable insights into ventilatory physiology during CPR, translating these findings to human clinical practice remains challenging. Despite their similarity to human cardiopulmonary physiology, differences in chest morphology and airway anatomy may influence effect of ventilation and its impact on gas exchange during resuscitation. Furthermore, controlled experimental settings only partially reflect the complexity of human cardiac arrest, including comorbidities and resuscitation quality. Beyond these biological and contextual differences, methodological heterogeneity in ventilatory management and monitoring further limits comparability between studies. By mapping current experimental practices, this scoping review highlights methodological gaps that, if properly considered, could improve validity and better delimit the translational value of animal research in CPR.

## Conclusion

Ventilatory settings and monitoring during experimental CPR in animals are reported with considerable variability and frequent omissions. Critical parameters such as PEEP, I:E ratio, inspiratory trigger (specific to mechanical ventilation), and EtCO_2_ analysis are often absent, highlighting the need for standardized reporting. Updating the minimal reporting standards would improve comparability, strengthen translational relevance, and guide the design of future animal studies.

## CRediT authorship contribution statement

**Jean-Claude Li:** Writing – review & editing, Writing – original draft, Formal analysis, Data curation, Conceptualization. **Nicolas Segond:** Writing – review & editing, Conceptualization. **Arnaud Lesimple:** Writing – review & editing, Writing – original draft, Visualization, Validation, Investigation, Formal analysis, Data curation, Conceptualization. **Alice Hutin:** Writing – review & editing, Writing – original draft, Visualization, Validation. **Rebecca Goutchtat:** Writing – review & editing, Visualization. **Iann Drennan:** Writing – review & editing, Conceptualization. **Giuseppe Ristagno:** Writing – review & editing, Conceptualization. **Guillaume Debaty:** Writing – review & editing, Conceptualization. **Alain Cariou:** Writing – original draft, Conceptualization. **Renaud Tissier:** Writing – review & editing, Writing – original draft, Visualization, Validation, Supervision, Project administration, Investigation, Funding acquisition, Formal analysis, Data curation, Conceptualization. **Jean-Christophe Richard:** Writing – review & editing, Writing – original draft, Visualization, Validation, Supervision, Project administration, Methodology, Investigation, Formal analysis, Data curation, Conceptualization.

## Ethics approval

This study did not properly involve the use of animals but only analyzed published investigations.

## Funding

The study was partly funded by the Agence Nationale pour la Recherche (Grant Areg-shock ANR-21-CE17-0011-03 and Grant CPR-CO2 ANR-25-CE19-4339-01).

## Declaration of competing interest

RT is shareholders of a start-up company dedicated to total liquid ventilation, which is not in the scope of the present work (Orixha). AL is medical engineer in the Med2Lab funded by Air Liquide Medical Systems. JCR reports part time salary for research activities (Med2Lab) from Air Liquide Medical Systems.

## Appendix A Supplementary material

Supplementary Data 1 **Supplemental Method:** Search strategy and **Supplemental Material:** List of the references analyzed in the scoping review.
