# Supplementary material for: Evaluation of ventilatory parameters reporting in large animal models of cardiac arrest: a scoping review
Source: Resusc Plus. 2025 Dec 8;27:101185. doi: 10.1016/j.resplu.2025.101185 (PMC12775993; doi:10.1016/j.resplu.2025.101185)
Supplement: Supplementary Data 1 — Supplemental Method: Search strategy and Supplemental Material: List of the references analyzed in the scoping review. [file mmc1.docx]

**Supplemental Method: Search strategy**

PubMed request:

*Animal selection:*

(Swine[MeSH] OR pig* OR porcine OR Dogs[MeSH] OR dog* OR canine OR Sheep[MeSH] OR sheep* OR ovine OR Goats[MeSH] OR goat* OR caprine OR lamb* OR "Primates, Non-Human"[MeSH] OR "non human primate*" OR monkey*) AND NOT (Humans[MeSH])

*Cardiac arrest or cardiopulmonary resuscitation:*

AND ("Cardiac Arrest"[MeSH] OR "Respiratory Arrest"[MeSH] OR "cardiac arrest" OR "cardiorespiratory arrest" OR "cardiopulmonary arrest" OR "Cardiopulmonary Resuscitation"[MeSH] OR "cardiopulmonary resuscitation" OR CPR)

*Ventilation monitoring and/or evaluation*

AND ("Respiration"[MeSH] OR "Ventilation"[MeSH] OR "respiratory parameters" OR "ventilatory parameters" OR "ventilatory support" OR "mechanical ventilation" OR (ventilatory settings) OR (ventilator) OR ventilation OR respiration)

*Period selection:*

AND ("2015/01/01"[PDAT]: "2025/07/30"[PDAT])

*Language selection:*

AND English[lang]

**Supplemental Material: List of the references analyzed in the scoping review**

*PMID, PubMed reference number assigned by the NIH National Library of Medicine*

| PMID | Author – Year - Title (first words) |
| --- | --- |
| 40355539 | Abbasi et al. - 2025 - Efficacy of laryngeal mask epinephrine in neonatal resuscitation; an ovine study |
| 38954057 | Abi Zeid Daou et al. - 2024 - Investigation of fingolimod-induced lymphocyte sequestration on inflammatory response and neurologic |
| 39158568 | Abi Zeid Daou et al. - 2024 - Ultrafast Cooling With Total Liquid Ventilation Mitigates Early Inflammatory Response and Offers Neu |
| 26690649 | Adams et al. - 2016 - Whole body periodic acceleration (pGz) preserves heart rate variability after cardiac arrest |
| 33839633 | Aggelina et al. - 2021 - Continuous chest compressions with asynchronous ventilation improve survival in a neonatal swine mod |
| 30820870 | Al-Subu et al. - 2020 - Two-site regional oxygen saturation and capnography monitoring during resuscitation after cardiac ar |
|  |  |
| 29042253 | Andreyev et al. - 2018 - Calcium uptake and cytochrome c release from normal and ischemic brain mitochondria |
| 30612967 | Babini et al. - 2019 - Effect of mild hypercapnia on outcome and histological injury in a porcine post cardiac arrest model |
| 39576879 | Badurdeen et al. - 2025 - Rapid oxygen titration following cardiopulmonary resuscitation mitigates cerebral overperfusion and |
| 35265564 | Berkelhamer et al. - 2022 - Inadequate Bioavailability of Intramuscular Epinephrine in a Neonatal Asphyxia Model |
| 38716382 | Bois et al. - 2024 - Impact of arginine-vasopressin on regional perfusions in a porcine model of post-resuscitation syndr |
| 26470011 | Cha et al. - 2015 - Comparison Between 301 and 302 Compression-to-ventilation Ratios for Cardiopulmonary Resuscitation |
| 37507472 | De La Mata Navazo et al. - 2023 - Volumetric capnography and return of spontaneous circulation in an experimental model of pediatric a |
| 27504455 | Faa et al. - 2016 - Effects of Erythropoietin Administration on Adrenal Glands of LandraceLarge White Pigs after Ventri |
| 34710880 | Fritz et al. - 2022 – Impact of different ventilation strategies on gas exchanges and circulation during prolonged mechani |
| 33289464 | Fumagalli et al. - 2020 - Ventilation With Argon Improves Survival With Good Neurological Recovery After Prolonged Untreated C |
| 38966232 | Gavriely et al. - 2024 - Novel gas mixture combined with an auto-transfusion tourniquet enhances cerebral O2 transport and he |
| 37726545 | Giusto et al. - 2024 - Neonatal resuscitation with continuous chest compressions and high frequency percussive ventilation |
| 32712173 | Hidalgo et al. - 2020 - Sustained inflation with 21% versus 100% oxygen during cardiopulmonary resuscitation of asphyxiated |
| 38971981 | Hoehne et al. - 2024 - Serial paired arterial and jugular venous point-of-care values in dogs undergoing manual basic life |
| 30327772 | Hopper et al. - 2018 - Efficacy of Manual Ventilation Techniques During Cardiopulmonary Resuscitation in Dogs |
| 26070832 | Hutin et al. - 2015 - Total liquid ventilation offers ultra-fast and whole-body cooling in large animals in physiological |
| 34002305 | Hutin et al. - 2021 - Resuscitative endovascular balloon occlusion of the aorta vs epinephrine in the treatment of non-tra |
| 39237256 | Jager et al. - 2025 - Evaluating the efficacy of endotracheal and intranasal epinephrine administration in severely asphyx |
| 26327555 | Jenkins et al. - 2015 - Effects of the ResQPOD on Kinetics, Hemodynamics of Vasopressin, and Survivability in a Porcine Card |
| 33539360 | Jung et al. - 2021 - Relationship of common hemodynamic and respiratory target parameters with brain tissue oxygen tensio |
| 26370446 | Kilbaugh et al. - 2015 - Persistently Altered Brain Mitochondrial Bioenergetics After Apparently Successful Resuscitation Fro |
| 26011525 | Kill et al. - 2015 - Chest Compression Synchronized Ventilation versus Intermitted Positive Pressure Ventilation during C |
| 30450858 | Kim et al. - 2018 - Single Ventilation during Cardiopulmonary Resuscitation Results in Better Neurological Outcomes in a |
| 34533046 | Kim et al. - 2021 - Comparison of Resuscitation Outcomes Between 2 or 3 Stacked Defibrillation Strategies With Minimall |
| 27165087 | Kjærgaard et al. - 2016 - Four ways to ventilate during cardiopulmonary resuscitation in a porcine model a randomized study |
| 32059732 | Kjaergaard et al. - 2020 - An impedance threshold device did not improve carotid blood flow in a porcine model of prolonged car |
| 40383501 | Ko et al. - 2025 - Attenuation of mitochondrial dysfunction in a ventricular fibrillation swine model of cardiac arrest |
| 31447395 | Kohlhauer et al. - 2020 - A new paradigm for lung-conservative total liquid ventilation |
| 36607514 | Kopra et al. - 2023 - Ventilation during continuous compressions or at 302 compression-to-ventilation ratio results in si |
| 40624444 | Kowal et al. - 2025 - Tidal volume delivery during chest compression with either an endotracheal tube or supraglottic airw |
| 32596995 | Kumar et al. - 2020 - Protection from systemic pyruvate at resuscitation in newborn lambs with asphyxial cardiac arrest |
| 31900987 | Lapid et al. - 2020 - The use of pressure-controlled mechanical ventilation in a swine model of intraoperative pediatric c |
| 30779720 | Lautz et al. - 2019 - Hemodynamic-Directed Cardiopulmonary Resuscitation Improves Neurologic Outcomes and Mitochondrial Fu |
| 31063840 | Lee et al. - 2019 - Oximetry-Guided normoxic resuscitation following canine cardiac arrest reduces cerebellar Purkinje n |
| 34308496 | Levenbrown et al. - 2021 - Effect of positive end-expiratory pressure on additional passive ventilation generated by CPR compre |
| 26766424 | Li et al. - 2016 - Exhaled CO2 Parameters as a Tool to Assess Ventilation-Perfusion Mismatching during Neonatal Resusci |
| 30498975 | Li et al. - 2019 - Identification of return of spontaneous circulation during cardiopulmonary resuscitation via pulse o |
| 30505723 | Liu et al. - 2018 - Quantitative CT assessment of lung injury after successful cardiopulmonary resuscitation in a porcin |
| 27618183 | López et al. - 2016 - Different Respiratory Rates during Resuscitation in a Pediatric Animal Model of Asphyxial Cardiac Ar |
| 29190801 | López et al. - 2017 - Comparison between manual and mechanical chest compressions during resuscitation in a pediatric anim |
| 32817703 | López et al. - 2020 - Effect of ventilation rate on recovery after cardiac arrest in a pediatric animal model |
| 28076627 | Macedo et al. - 2016 - PetCO2, VCO2 and CorPP Values in the Successful Prediction of the Return of Spontaneous Circulation |
| 39382715 | Magliocca et al. - 2024 - A multimodal characterization of cardiopulmonary resuscitation-associated lung edema |
| 37938394 | Mälberg et al. - 2023 - Continuous chest compressions are associated with higher peak inspiratory pressures when compared to |
| 31318890 | Manrique et al. - 2019 - Comparison between synchronized and non-synchronized ventilation and between guided and non-guided c |
| 34373497 | Manrique et al. - 2021 - Effects of airway management and tidal volume feedback ventilation during pediatric resuscitation in |
| 28408349 | Matsuura et al. - 2017 - Early Effects of Prolonged Cardiac Arrest and Ischemic Postconditioning during Cardiopulmonary Resus |
| 36979878 | Mohnke et al. - 2023 - The Influence of Ultra-Low Tidal Volume Ventilation during Cardiopulmonary Resuscitation on Renal an |
| 38006467 | Mohnke et al. - 2023 - Ultra-low tidal volume ventilation during cardiopulmonary resuscitation shows no mitigating effect o |
| 39048668 | Morin et al. - 2024 - Cardiopulmonary resuscitation with 31 CompressionVentilation or continuous compression with asynch |
| 38617441 | Morin et al. - 2024 - Continuous chest compression during sustained inflation versus continuous compression with asynchron |
| 29928955 | Mustofa et al. - 2018 - Effects of different durations of sustained inflation during cardiopulmonary resuscitation on return |
| 28438718 | Nelskylä et al. - 2017 - The effect of 50% compared to 100% inspired oxygen fraction on brain oxygenation and post cardiac ar |
| 36470536 | Nelskylä et al. - 2023 - The use of 100% compared to 50% oxygen during ineffective experimental cardiopulmonary resuscitation |
| 34223293 | Neumann et al. - 2020 - First-time evaluation of ascending compared to rectangular transthoracic defibrillation waveforms in |
| 38155976 | Olasveengen et al. - 2023 - Lung tissue injury and hemodynamic effects of ventilations synchronized or unsynchronized to continu |
| 26423768 | Ordelman et al. - 2015 - A ventilation technique for oxygenation and carbon dioxide elimination in CPR Continuous insufflati |
| 37585896 | Palácio et al. - 2023 - Use of a Portable Mechanical Ventilator during Cardiopulmonary Resuscitation is Feasible, Improves R |
| 39040822 | Persson et al. - 2024 - Hypothermia After Cardiac Arrest in Large Animals (HACA-LA) Study protocol of a randomized controll |
| 32848852 | Polglase et al. - 2020 - Cardiopulmonary Resuscitation of Asystolic Newborn Lambs Prior to Umbilical Cord Clamping; the Timin |
| 38123977 | Polglase et al. - 2024 - Assessing the influence of abdominal compression on time to return of circulation during resuscitati |
| 38522732 | Polglase et al. - 2024 - Endotracheal epinephrine at standard versus high dose for resuscitation of asystolic newborn lambs |
| 39029577 | Pourzand et al. - 2024 - Active decompression during automated head-up cardiopulmonary resuscitation |
| 38043854 | Pourzand et al. - 2024 - Hemodynamics, survival and neurological function with early versus delayed automated head-up CPR in |
| 39970974 | Pourzand et al. - 2025 - Intraventricular pressure and volume during conventional and automated head-up CPR |
| 39104445 | Power et al. - 2024 - Feasibility of a 4 French resuscitative endovascular balloon occlusion of the aorta (REBOA) device f |
| 27095125 | Putzer et al. - 2016 - Monitoring of brain oxygenation during hypothermic CPR – A prospective porcine study |
| 30987091 | Rawat et al. - 2019 - Oxygenation and Hemodynamics during Chest Compressions in a Lamb Model of Perinatal Asphyxia Induced |
| 36832479 | Rawat et al. - 2023 - Masked Randomized Trial of Epinephrine versus Vasopressin in an Ovine Model of Perinatal Cardiac Arr |
| 36013161 | Renz et al. - 2022 - High PEEP Levels during CPR Improve Ventilation without Deleterious Haemodynamic Effects in Pigs |
| 37790622 | Renz et al. - 2023 - Analysis of cerebral Interleukin-6 and tumor necrosis factor alpha patterns following different vent |
| 31442471 | Roh et al. - 2019 - Shorter defibrillation interval promotes successful defibrillation and resuscitation outcomes |
| 37494389 | Roh et al. - 2023 - Development of an automatic device performing chest compression and external defibrillation An anim |
| 30176273 | Ruemmler et al. - 2018 - Ultra-low tidal volume ventilation—A novel and effective ventilation strategy during experimental ca |
| 32377456 | Ruemmler et al. - 2020 - Bi-Level ventilation decreases pulmonary shunt and modulates neuroinflammation in a cardiopulmonary |
| 39962856 | Rysz et al. - 2025 - Levosimendan improves central haemodynamic status and gas exchange in a model of ischaemic cardiac a |
| 29649316 | Ryu et al. - 2018 - Effect of one-lung ventilation on end-tidal carbon dioxide during cardiopulmonary resuscitation in a |
| 27529347 | Sachse et al. - 2016 - The Role of Plasma and Urine Metabolomics in Identifying New Biomarkers in Severe Newborn Asphyxia |
| 34763236 | Salcido et al. - 2022 - Injury characteristics and hemodynamics associated with guideline-compliant CPR in a pediatric porci |
| 34205843 | Sankaran et al. - 2021 - Effect of a Larger Flush Volume on Bioavailability and Efficacy of Umbilical Venous Epinephrine duri |
| 33687959 | Sankaran et al. - 2021 - Randomised trial of epinephrine dose and flush volume in term newborn lambs |
| 33941864 | Sankaran et al. - 2021 - Randomized trial of oxygen weaning strategies following chest compressions during neonatal resuscita |
| 34284596 | Schmölzer et al. - 2021 - Sustained Inflation During Chest Compression A New Technique of Pediatric Cardiopulmonary Resuscita |
| 34844983 | Schmölzer et al. - 2022 - Single versus continuous sustained inflations during chest compressions and physiological-based cord |
| 26761124 | Seehase et al. - 2016 - Propofol administration to the fetal–maternal unit reduces cardiac oxidative stress in preterm lambs |
| 32574159 | Shim et al. - 2020 - Effects of sustained inflation pressure during neonatal cardiopulmonary resuscitation of asphyxiated |
| 34223383 | Smida et al. - 2021 - Early cytotoxic lymphocyte localization to the brain following resuscitation in a porcine model of a |
| 30862527 | Soltesz et al. - 2019 - Phase-controlled intermittent intratracheal insufflation of oxygen during chest compression-active d |
| 28187154 | Tan et al. - 2017 - Comparison of different inspiratory triggering settings in automated ventilators during cardiopulmon |
| 31183772 | Tan et al. - 2020 - Duration of cardiac arrest requires different ventilation volumes during cardiopulmonary resuscitati |
| 34223377 | Udassi et al. - 2021 - Chest compression by two-thumb encircling method generates higher carotid artery blood flow in swine |
| 28661972 | Vali et al. - 2017 - Continuous Chest Compressions During Sustained Inflations in a Perinatal Asphyxial Cardiac Arrest La |
| 28214793 | Vali et al. - 2017 - Evaluation of Timing and Route of Epinephrine in a Neonatal Model of Asphyxial Arrest |
| 29244793 | Wagner et al. - 2018 - Effects of epinephrine on hemodynamic changes during cardiopulmonary resuscitation in a neonatal pig |
| 40034872 | Wagner et al. - 2025 - Adrenaline has a limited effect on myocardial microvascular blood flow A randomised experimental st |
| 34984109 | Wang et al. - 2022 - Evaluation of a Novel Left Ventricular Assist Device for Resuscitation in an Animal Model of Ventric |
| 28098197 | Wiesmann et al. - 2017 - Dantrolene versus amiodarone for cardiopulmonary resuscitation a randomized, double-blinded experim |
| 28915219 | Wolf et al. - 2018 - Intrabronchial Catheter Resuscitation for Respiratory and Cardiorespiratory Arrest |
| 32220582 | Xu et al. - 2020 - Modified volumetric capnography-derived parameter A potentially stable indicator in monitoring card |
| 36619612 | Xu et al. - 2022 - The combination of chest compression synchronized ventilation and aortic balloon occlusion improve t |
| 29221300 | Yang et al. - 2017 - Comparison of continuous compression with regular ventilations versus 302 compressions-ventilations |
| 28584212 | Yang et al. - 2017 - Variations of Postresuscitation Lung Function after Thrombolysis Therapy in a Cardiac Arrest Porcine |
| 31549565 | Yang et al. - 2019 - Alterations in Respiratory Mechanics and Neural Respiratory Drive After Restoration of Spontaneous C |
| 34277042 | Zhang et al. - 2021 - New volumetric capnography-derived parameter a potentially valuable tool for detecting hyperventila |
| 33511562 | Zhang et al. - 2022 - Assessment of a new volumetric capnography-derived parameter to reflect compression quality and to p |
